# Clinical and microbiological characteristics and outcomes of community-acquired sepsis among adults: a single center, 1-year retrospective observational cohort study from Hungary

**DOI:** 10.1186/s12879-019-4219-5

**Published:** 2019-07-26

**Authors:** Balint Gergely Szabo, Rebeka Kiss, Katalin Szidonia Lenart, Bence Marosi, Eszter Vad, Botond Lakatos, Eszter Ostorhazi

**Affiliations:** 1 Semmelweis University, School of PhD Studies, H-1085 Ulloi ut 26., Budapest, Hungary; 2 Department of Infectious Diseases, South Pest Central Hospital, National Institute of Hematology and Infectious Diseases, Saint Ladislaus Campus, H-1097 Albert Florian ut 5-7., Budapest, Hungary; 3 Infectious Disease Specialist Training, Semmelweis University, Faculty of Medicine, H-1085 Ulloi ut 26., Budapest, Hungary; 4 Department of Infectious Diseases, Markhot Ferenc Teaching Hospital, H-3300 Szechenyi utca 27-29., Eger, Hungary; 5 Faculty of Medicine, Students’ Scientific Association, Semmelweis University, H-1085 Ulloi ut 26., Budapest, Hungary; 6 South Pest Central Hospital, National Institute of Hematology and Infectious Diseases, Saint Ladislaus Campus, Core Microbiology Laboratory, H-1097 Albert Florian ut 5-7., Budapest, Hungary; 7 Semmelweis University, Institute of Medical Microbiology, H-1098 Nagyvarad ter 4., Budapest, Hungary

**Keywords:** Infection, SIRS, Sepsis, Septic shock, Community-acquired, Mortality

## Abstract

**Background:**

Community-acquired sepsis is a life-threatening systemic reaction, which starts within ≤72 h of hospital admittance in an infected patient without recent exposure to healthcare risks. Our aim was to evaluate the characteristics and the outcomes concerning community-acquired sepsis among patients admitted to a Hungarian high-influx national medical center.

**Methods:**

A retrospective, observational cohort study of consecutive adult patients hospitalized with community-acquired sepsis during a 1-year period was executed. Clinical and microbiological data were collected, patients with pre-defined healthcare associations were excluded. Sepsis definitions and severity were given according to *ACCP*/*SCCM* criteria. The primary outcome was in-hospital all-cause mortality. Secondary outcomes were intensive care unit (ICU) admittance, length-of-stay (LOS), source control and bacteraemia rates. Statistical differences were explored with classical comparison tests, predictors of in-hospital all-cause mortality were modelled by multivariate logistic regression.

**Results:**

214 patients (median age 60.0 ± 33.1 years, 57% female, median Charlson score 4.0 ± 5.0) were included, 32.7% of them (70/214) had severe sepsis, and 28.5% (61/214) had septic shock. Prevalent sources of infections were genitourinary (53/214, 24.8%) and abdominal (52/214, 24.3%). The causative organisms were dominantly *E. coli* (60/214, 28.0%), *S. pneumoniae* (18/214, 8.4%) and *S. aureus* (14/214, 6.5%), and bacteraemia was documented in 50.9% of the cases (109/214). In-hospital mortality was high (30/214, 14.0%), and independently associated with shock, absence of fever, male gender and the need for ICU admittance, but source control and de-escalation of empirical antimicrobial therapy were protective. ICU admittance was 27.1% (58/214), source control was achieved in 18.2% (39/214). Median LOS was 10.0 ± 8.0, ICU LOS was 8.0 ± 10.8 days.

**Conclusions:**

Community-acquired sepsis poses a significant burden of disease with characteristic causative agents and sources. Patients at a higher risk for poor outcomes might be identified earlier by the contributing factors shown above.

## Background

Sepsis is a dysregulated host response given to an infection, which ultimately leads to systemic inflammation, organ dysfunction or death [1–3]. Data from current literature suggest that the incidence of sepsis steadily increased during the past four decades in high-income countries [4, 5]. This alarming trend might be explained by the growing proportions of immunocompromised and elderly patients living with a significant comorbidity burden, as well as infections caused by multi-resistant organisms, and possibly increased recognition of warning signs by physicians. Despite an earlier detection and the advances in clinical strategies including sepsis-oriented intensive care support, the mortality of sepsis is still considerably high [6]. Therefore, sepsis is still one of the leading causes of death among patients with infectious diseases, and was designated as a medical priority by the *Centers for Disease Control and Prevention* (CDC), the *World Health Assembly* and the *World Health Organization* [7, 8].

Community-acquired sepsis (CAS) starts within 48–72 h after hospital admittance in a patient without recently documented connections to healthcare facilities or modalities. According to data collected by the CDC, approximately 80% of all sepsis cases start outside the hospital, and 7 out of 10 patients suffer from chronic illnesses [7]. It is estimated that 20–40% of these septic patients admitted from the community require further treatment in an intensive care unit (ICU), and in-hospital mortality could be as high as 25–60%, but more current data concerning CAS, especially among Hungarian patients are required [9–12]. Therefore our aim was to evaluate the epidemiological, the microbiological characteristics and the clinical outcomes of CAS among adult patients admitted to our tertiary referral center.

## Methods

### Study design and population

A retrospective, observational cohort study was carried out by analysing cases of consecutive adult (age ≥ 18 years at diagnosis) patients with community-acquired sepsis admitted to South Pest Central Hospital, National Institute of Hematology and Infectious Diseases (Budapest, Hungary) between January 1st, 2016 and December 31st, 2016. Our tertiary referral center hosts a high-influx, 140-bed department of infectious diseases with nationwide coverage. The study protocol was in accordance with the Helsinki Declaration and the international ethical standards, and has been approved by the institutional ethical board. Obtaining informed consent is not required for this type of study.

### Patient identification and inclusion

All patients were eligible for inclusion if they were diagnosed with any severity of sepsis at our center during the study period. Patient identification was performed by searching through the hospital electronical database for admittance and discharge diagnoses consistent with sepsis, severe sepsis or septic shock, as given by the *International Classification of Diseases* (ICD–10), 10th edition (A02.1–02.9, A39.0–39.9, A40.0–40.9, A41.0–41.9, A42.70, A48.0–48.8, A49.0–49.9, R57.1–57.9). To overcome selectional bias, all potential cases were manually evaluated. Patients were included if they were admitted with signs and symptoms consistent with infection, their case satisfied SIRS based sepsis criteria (see “Definitions”), and if the time to sepsis onset or diagnosis was within ≤72 h from admittance or transportation occured from another healthcare facility or the community within this time frame to our center because of CAS. Patients fulfilling any of the pre-defined criteria listed were excluded: 1) onset or diagnosis > 72 h from admittance, 2) sepsis due to nosocomial infection during hospital stay, 3) hospitalization at any healthcare facility for ≥72 h, systemic antimicrobial treatment or cavity-opening surgery within 90 days pre-admittance, 4) regular outpatient visits (iv. therapy or chemotherapy, chronic wound care, hemodialysis) within 30 days pre-admittance, 5) long-term care facility residence for ≥3 days, 6) healthcare worker, 7) data inaccessible through the hospital electronic database.

### Definitions

A pathogen was accepted as a causative organism of sepsis if it was microbiologically identified from a clinically relevant sample during a compatible case presentation. Identification of causative organisms was accomplished by classical bacterial and fungal culturing methods and matrix-assisted laser desorption/ionization time-of-flight mass spectrometry (MALDI/TOF–MS), as well as using non-culture based methods such as in vitro antigen detection (for *S. pneumoniae, L. pneumophila, H. influenzae, N. meningitidis*), in vitro toxin detection, (for *C. difficile*), serology (for *M. pneumoniae, C. pneumoniae, L. pneumophila*) and PCR (for *S. pneumoniae, L. pneumophila, H. influenzae, N. meningitidis, L. monocytogenes,* West Nile virus, EBV, CMV, HSV1) techniques, as deemed appropriate. A multidrug-resistant (MDR) bacterium was defined as an isolate with acquired non-susceptibility to ≥1 tested antimicrobial agents in ≥3 antimicrobial classes, as defined extensively by *Magiorakos* et al. [13]. Microbiological tests were carried out at the Core Microbiology Laboratory of South Pest Central Hospital, National Institute of Hematology and Infectious Diseases. Sepsis, severe sepsis and septic shock were defined by the SIRS based adult criteria of *American College of Chest Physicians/Society of Critical Care Medicine* [1, 2]. The Charlson comorbidity score was calculated for each patient [14]. Fever was defined as a single tympanal temperature of ≥38.0 °C measured with an electronic thermometer before administration of antipyretics or antimicrobials. Empiric antibiotic therapy was considered adequate if: 1) the patient received at least one initial antibiotic within 1 h of sepsis recognition for the suspected source in the dose range and administration route recommended by current international standards pending microbiological results, 2) escalation was not required and 3) in vitro susceptibility of the causative agent(s) was later documented, or there was no presumable intrinsic resistance if the causative agent was demonstrated by non-culture based tests only. Empirical antibiotic therapy was considered inadequate if any of these criteria were not fulfilled. At our centre, early recognition and management, including adequate choices and timing of empiric antimicrobial therapy, is facilitated by a sepsis-oriented, standardized institutional protocol. Escalation was defined if the empirical antimicrobial choice was altered or switched pending microbiological results to broaden the antimicrobial spectrum. Targeted antibiotic therapy was defined as continuation or narrowing of the empirical regime (de-escalation) after the acknowledgment of microbiological results. Antibiotics were grouped separately into their respective pharmacological classes for comparisons. A supportive therapy was identified as ≥1 of the following for ≥24 h initiated due to CAS: 1) mechanical ventilation, 2) hemodynamical support, 3) renal replacement therapy, 4) human albumin or blood product substitution, 5) corticosteroid therapy.

### Data collection, follow-up and patient outcomes

The following data were collected: 1) age, gender, and comorbidites known at diagnosis, 2) progression of sepsis (onset time, severity and source of sepsis, symptoms and results of physical examination at diagnosis), 3) laboratory findings at diagnosis, 4) microbiological findings, 5) characteristics of antimicrobial and supportive therapies, and6) patient outcomes. Data were anonymously collected and transferred to a standardized, electronic case report form. Patients were followed up for their total hospitalization time by using the database, but post-discharge follow up was not carried out. The primary outcome was in-hospital all-cause mortality, secondary outcomes were ICU admittance, length-of-stay (LOS and ICU LOS), source control and bacteraemia rates. Characteristics of causative agents, sources of sepsis, types and durations of antimicrobial and supportive therapies were also evaluated.

### Desciption of statistical methods

We express continuous variables as median ± interquartile region (IQR) with minimum–maximum intervals. For two-sample comparisons, *Student*’s t-test and *Mann–Whitney* U-test, for multiple comparisons, ANOVA and *Kruskal–Wallis* test with *Bonferroni*’s post hoc correction were used. We express categorical values as absolute numbers (n) with percentages (%), statistical comparisons were made by *Fisher*’s exact test and *Pearson*’s χ^2^ test. For the estimation of annual incidence, the adult population data for our total referral area were used from the last nationwide census [15]. For the identification of clinical and microbiological risk factors independently associating with in-hospital mortality, uni- and multivariate logistic regression was executed. All biologically plausible parameters and those with a *p* value of ≤0.1 in univariate analysis were entered into a forward stepwise multivariate logistic regression (entry criterion: *p* = 0.05, removal criterion: *p* = 0.1). For testing the goodness of model fit, the *Hosmer–Lemeshow* test was used. Statistical significance was determined at a two-tailed *p* value of < 0.05. Data collection was completed with Microsoft Office Excel 2016; calculations were done using GraphPad Prism 5 and IBM SPSS Statistics 23.

## Results

### Demographic and clinical characteristics

Demographic and clinical data are shown in Table 1. During the study period, 629 eligible cases were identified, and from these, 214 (34.0%) were included in our study, 38.8% (83/214) with sepsis, 32.7% (70/214) with severe sepsis and 28.5% (61/214) with septic shock. The estimated annual incidence corresponded to 7.2 cases per 100,000 inhabitants, translating to 2.96% of hospital admittances. In the cohort, 23.8% (51/214) of patients were ≥ 75 years old with equally distributed genders, and 57.0% (122/214) had a Charlson score of ≥4, with arterial hypertension (132/214, 61.7%), chronic heart disease (94/214, 43.9%,) and diabetes mellitus (61/214, 28.5%) being the most prevalent comorbidities. Patients with septic shock had significantly higher rates of chronic liver (15.7% vs. 34.4%, *p* = 0.02) and cerebrovascular (18.6% vs. 37.7%, *p* = 0.04) diseases, compared to sepsis and severe sepsis, respectively. In addition, severe sepsis was frequently associated with corticosteroid usage (2.4% vs. 15.7%, *p* = 0.01). Leading symptoms at onset were fever (192/214, 89.7%), hypotension and tachycardia (135/214, 63.1% each), and clinical signs of hemodynamical and respiratory instability associated with septic shock. The prevalent sources of sepsis were genitourinary (53/214, 24.8%) and abdominal infections (52/214, 24.3%), but an obvious source could not be identified in 11.2% (24/214) of the cases. Septic shock mainly arose from abdominal infections (15.7% vs. 37.7%, *p* = 0.01).

**Table 1:** Demographical and clinical data of adult patients with community-acquired sepsis by severity group

Parameters^a^	Total (*n* = 214)	Sepsis (*n* = 83)	Severe sepsis (*n* = 70)	Septic shock (*n* = 61)	*p* value
Age (years, median ± IQR, min–max)	60.0 ± 33.1 (18–93)	58.5 ± 26.5 (20–92)	61.3 ± 36.5 (18–95)	62.0 ± 26.5 (25–90)	0.58
Male gender	92 (43.0)	31 (37.3)	34 (48.6)	27 (44.3)	0.36
Comorbidities:					
- Arterial hypertension	132 (61.7)	51 (61.4)	44 (62.9)	37 (60.7)	0.96
- Chronic heart disease	94 (43.9)	30 (36.1)	34 (48.6)	30 (49.2)	0.18
- Chronic lung disease	34 (15.9)	11 (13.3)	12 (17.1)	11 (18.0)	0.69
- Chronic kidney disease	50 (23.4)	15 (18.1)	21 (30.0)	14 (23.0)	0.22
- Chronic liver disease	55 (25.7)	13 (15.7)	21 (30.0)	21 (34.4)	0.02*
- Diabetes mellitus	61 (28.5)	24 (28.9)	24 (34.3)	13 (21.3)	0.26
- Malignancy	22 (10.3)	11 (13.3)	6 (8.6)	5 (8.2)	0.52
- Chronic corticosteroid use	17 (7.9)	2 (2.4)	11 (15.7)	4 (6.6)	0.01**
- Immunosuppression	57 (26.6)	18 (21.7)	24 (34.3)	15 (24.6)	0.19
- Cerebrovascular disease	58 (27.1)	22 (26.5)	13 (18.6)	23 (37.7)	0.04***
- Intravenous drug use	7 (3.3)	2 (2.4)	4 (5.7)	1 (1.6)	0.36
- Excess alcohol use	45 (21.0)	13 (15.7)	13 (18.6)	19 (31.1)	0.06
No. of comorbidities (per patient, median ± IQR, min–max)	3.0 ± 3.0 (0–8)	2.0 ± 3.0 (0–7)	3.5 ± 4.0 (0–8)	3.0 ± 3.0 (0–7)	0.07
Charlson score (median ± IQR, min–max)	4.0 ± 5.0 (0–12)	3.0 ± 5.0 (0–10)	4.5 ± 5.0 (0–12)	5.0 ± 5.0 (0–10)	0.17
Source of sepsis:					
Head or neck source	29 (13.6)	13 (15.7)	10 (14.3)	6 (9.8)	0.58
- Meningitis	24 (11.2)	10 (12.0)	8 (11.4)	6 (9.8)	
- Tonsillopharyngitis	4 (1.9)	3 (3.6)	1 (1.4)	0	
- Otitis media	1 (0.5)	0	1 (1.4)	0	
Thoracic source	30 (14.0)	11 (13.3)	10 (14.3)	9 (14.8)	0.96
- Pneumonia	28 (13.1)	10 (12.0)	9 (12.9)	9 (14.8)	
- Endocarditis	2 (0.9)	1 (1.2)	1 (1.4)	0	
Abdominal source	52 (24.3)	13 (15.7)	16 (22.9)	23 (37.7)	0.01*
- Peritonitis	12 (5.6)	2 (2.4)	1 (1.4)	9 (14.8)	
- Cholangitis	19 (8.9)	5 (6.0)	8 (11.4)	6 (9.8)	
- Colonic perforation	2 (0.9)	0	0	2 (3.3)	
- Oesophagitis	1 (0.5)	1 (1.2)	0	0	
- Enterocolitis	18 (8.4)	5 (6.0)	7 (10.0)	6 (9.8)	
Skin and soft tissue source	20 (9.3)	8 (9.6)	5 (7.1)	7 (11.5)	0.69
- Cellulitis	17 (7.9)	6 (7.2)	4 (5.7)	7 (11.5)	
- Diabetic foot infection	3 (1.4)	2 (2.4)	1 (1.4)	0	
Urogenital source	53 (24.8)	26 (31.3)	18 (25.7)	9 (14.8)	0.07
- Urinary tract infection	50 (23.4)	23 (27.7)	18 (25.7)	9 (14.8)	
- Pelvic inflammatory disease	3 (1.4)	3 (3.6)	0	0	
Toxic shock syndrome	6 (2.8)	0	2 (2.9)	4 (6.6)	0.06
Unknown source	24 (11.2)	12 (14.5)	9 (12.9)	3 (4.9)	0.17
Signs of sepsis:					
- Fever	192 (89.7)	74 (89.2)	66 (94.3)	52 (85.2)	0.23
- Hypothermia	2 (0.9)	0	2 (2.9)	0	n.a.
- Euthermia	21 (9.8)	9 (10.8)	3 (4.3)	9 (14.8)	0.12
- Hypotension	135 (63.1)	27 (32.5)	47 (67.1)	61 (100.0)	< 0.01*^,^**^,^***
- Tachycardia	135 (63.1)	33 (39.8)	46 (65.7)	56 (91.8)	< 0.01*^,^**^,^***
- Tachypnea	90 (42.1)	16 (19.3)	36 (51.4)	38 (62.3)	< 0.01*^,^**
- Oliguria	69 (32.2)	6 (7.2)	22 (31.4)	41 (67.2)	< 0.01*^,^**^,^***
- Altered mental status	84 (39.3)	16 (19.3)	25 (35.7)	43 (70.5)	< 0.01*^,^***
- Acute abdomen	26 (12.1)	7 (8.4)	8 (11.4)	11 (18.0)	0.18
- Bleeding	18 (8.4)	3 (3.6)	3 (4.3)	12 (19.7)	0.01*^,^***
- Skin lesion	56 (26.2)	16 (19.3)	16 (22.9)	24 (39.3)	0.02*
- Peripheral oedema	41 (19.2)	10 (12.0)	13 (18.6)	18 (29.5)	0.03*
- Splenomegaly	50 (23.4)	16 (19.3)	17 (24.3)	17 (27.9)	0.47

^a^ All parameters were documented at diagnosis of sepsis, and data are reported in absolute numbers and relative percentages (n, %), unless otherwise specified

*n.a.* Not applicable.

* *p* < 0.05 for comparison between the groups sepsis and septic shock

** *p* < 0.05 for comparison between the groups sepsis and severe sepsis

*** *p* < 0.05 for comparison between the groups severe sepsis and septic shock

### Microbiological characteristics

Microbiological characteristics are described in Table 2*.* Blood cultures were taken in 92.5% (198/214) of cases. Additionally, 71.5% (153/214) of patients had other relevant samples collected as well, and non-culture based diagnostic tests were applied in 20.6% (44/214). The dominance of Gram negative bacilli was observed in the cohort, with *E. coli* (60/214, 28.0%) being the most common pathogen, followed by *K. pneumoniae* (18/214, 8.4%), *S.* Enteritidis (10/214, 4.7%) and other Gram negative rods (15/214, 7.0%). Among Gram positive pathogens, *S. pneumoniae* (18/214, 8.4%), *S. aureus* (14/214, 6.5%) and *S. pyogenes* (8/214, 3.7%) were detected frequently. Obligate anaerobes were isolated in 1.9% (4/214), and 1.4% (3/214) of pathogens were viral or fungal. The causative organism could not be identified in 25.7% (55/214), and in 3.3% (7/214), sepsis was polymicrobial. No statistically significant differences in frequencies of causative agents were noted between subgroups of severity. *S. pneumoniae* was the most common pathogen among head–neck and thoracic sources, *E. coli* was prevalent in abdominal and genitourinary infections, while *S. aureus* dominated among skin and sofft tissue infections. Only 4/214 (1.9%) cases were caused by MDR isolates: 3/214 (1.4%) by ESBL producing *E. coli* and 1/214 (0.5%) by methicillin-resistant *S. aureus* (MRSA). Acquired carbapenem resistance was not detected among Gram negative isolates.

**Table 2:** Microorganisms identified as causative agents in adult patients with community-acquired sepsis by severity group

Pathogens^a^	Total (n = 214)	Sepsis (n = 83)	Severe sepsis (n = 70)	Septic shock (n = 61)	*p* value
*E. coli*	60 (28.0)	25 (30.1)	21 (30.0)	14 (23.0)	0.89
*S. pneumoniae*	18 (8.4)	4 (4.8)	6 (8.6)	8 (13.1)	0.79
*S. aureus*	14 (6.5)	7 (8.4)	3 (4.3)	4 (6.6)	0.99
*K. pneumoniae*	12 (5.6)	2 (2.4)	5 (7.1)	5 (8.2)	0.98
*S.* Enteritidis	10 (4.7)	2 (2.4)	5 (7.1)	3 (4.9)	1.0
*S. pyogenes*	8 (3.7)	2 (2.4)	3 (4.3)	3 (4.9)	1.0
*N. meningitidis*	5 (2.3)	2 (2.4)	2 (2.9)	1 (1.6)	1.0
*L. monocytogenes*	3 (1.4)	1 (1.2)	1 (1.4)	1 (1.6)	1.0
*K. oxytoca*	3 (1.4)	0	1 (1.4)	2 (3.3)	1.0
*P. mirabilis*	3 (1.4)	0	2 (2.9)	1 (1.6)	1.0
*S. anginosus*	2 (0.9)	0	1 (1.4)	1 (1.6)	1.0
*C. jejuni*	2 (0.9)	0	0	2 (3.3)	n.a.
*L. pneumophila*	2 (0.9)	0	1 (1.4)	1 (1.6)	1.0
*S. agalactiae*	2 (0.9)	2 (2.4)	0	0	n.a.
*F. necrophorum*	2 (0.9)	2 (2.4)	0	0	n.a.
West Nile virus	2 (0.9)	2 (2.4)	0	0	n.a.
*S. mitis*	2 (0.9)	2 (2.4)	0	0	n.a.
*S. dysgalactiae*	1 (0.5)	1 (1.2)	0	0	n.a.
*C. difficile*	1 (0.5)	0	0	1 (1.6)	n.a.
*P. aeruginosa*	1 (0.5)	0	0	1 (1.6)	n.a.
*B. thetaiotamicron*	1 (0.5)	0	0	1 (1.6)	n.a.
*C. albicans*	1 (0.5)	1 (1.2)	0	0	n.a.
*C. canimorsus*	1 (0.5)	0	0	1 (1.6)	n.a.
*E. cloaceae*	1 (0.5)	1 (1.2)	0	0	n.a.
*P. multocida*	1 (0.5)	1 (1.2)	0	0	n.a.
*S. marcescens*	1 (0.5)	0	1 (1.4)	0	n.a.

^a^All data are reported in absolute numbers and relative percentages (n, %)

*n.a.* Not applicable.

### Antimicrobial and supportive therapies

Characteristics of antimicrobial and supportive treatment are described in Table 3. The most frequently initiated empirical antimicrobial agent was ceftriaxone (140/212, 66.0%), followed by vancomycin (20/212, 9.4%) and levofloxacin (14/212, 6.6%). In contrast, the empirical use of piperacillin-tazobactam (5/212, 2.4%), imipenem-cilastatin (10/212, 4.7%) and meropenem (5/212, 2.4%) were infrequent. A combination of two or three antimicrobials was favoured among septic shock patients (20/60, 33.3%). Penicillins for sepsis (18.3% vs. 4.3%, *p* = 0.02) and carbapenems for septic shock (1.2% vs. 16.7%, *p* = 0.01) were administered in significantly higher percentages, compared to severe sepsis and sepsis, respectively. The time from appearance of first symptoms consistent with CAS to the first antibiotic dose was 3.0 ± 2.7 (1–6) days, but 74.1% (157/212) of patients received the first dose within 24 h of symptom onset. Empirical antibiotic therapy was deemed adequate in 89.6% (190/212), the need for escalation was 16.9% (36/212) altogether with imipenem-cilastatin (11/36, 30.6%) as the drug of first choice. De-escalation of initial therapy was performed in 14.2% (30/212), mostly in septic shock (14.6% vs. 23.3%, p = 0.02). Ceftriaxone (26/30, 86.7%) was preferred for de-escalation. In 25.9% (55/212) of cases, intravenous medication could be exchanged to an oral alternative, typically to ciprofloxacin (24/55, 43.6%), cefuroxime (12/55, 21.8%) and cefixime (6/55, 10.9%). Duration of antimicrobial therapy was 10.0 ± 5.0 (1–50) days. Supportive therapy was necessary in 40.7% (87/214) of cases, mostly among patients with septic shock. Noradrenaline (28/47, 59.6%) was the most prevalently administered vasopressor, followed by dopamine (17/47, 36.2%) and terlipressin (2/47, 4.3%).

**Table 3:** Clinical outcomes, antimicrobial and supportive treatment of adult patients with community-acquired sepsis by severity group

Parameters^a^	Total (n = 214)	Sepsis (n = 83)	Severe sepsis (n = 70)	Septic shock (n = 61)	*p* value
In-hospital mortality	30 (14.0)	0	4 (5.7)	26 (42.6)	< 0.01**^,^***
Time to death from admission (days, median ± IQR, min–max)	8.0 ± 12.5 (1–40)	0	5.0 ± 8.8 (2–19)	7.0 ± 10.8 (1–40)	0.6
ICU admittance	58 (27.1)	7 (8.4)	16 (22.9)	35 (57.4)	< 0.01*^,^**^,^***
LOS (days, median ± IQR, min–max)	10.0 ± 8.0 (1–123)	10.0 ± 7.5 (3–80)	10.0 ± 6.0 (1–123)	7.0 ± 13.8 (1–80)	0.96
ICU LOS (days, median ± IQR, min–max)	8.0 ± 10.8 (1–60)	9.0 ± 14.5 (2–22)	9.0 ± 7.0 (1–50)	8.0 ± 11.0 (1–60)	0.95
Rate of source control	39 (18.2)	13 (15.7)	13 (18.6)	13 (21.3)	0.76
Rate of bacteraemia	109 (50.9)	37 (44.6)	40 (57.1)	32 (52.5)	0.29
No. of patients receiving antimicrobial therapy	212 (99.1)	82 (98.8)	70 (100)	60 (98.4)	0.44
Duration of antibiotic therapy^b^ (days, median ± IQR, min–max)	10.0 ± 5.0 (1–50)	10.5 ± 4.0 (4–50)	10.0 ± 5.0 (2–28)	12.5 ± 13.0 (1–30)	0.61
Types of empirical antimicrobial therapy:					
- Penicillins	26 (12.3)	15 (18.3)	3 (4.3)	8 (13.3)	0.02*
- Cephalosporins	153 (72.2)	56 (68.3)	55 (78.6)	42 (70.0)	0.7
- Carbapenems	16 (7.6)	1 (1.2)	5 (7.1)	10 (16.7)	0.01**
- Aminoglycosides	5 (2.4)	4 (4.8)	1 (1.4)	0	0.99
- Macrolides	1 (0.5)	0	0	1 (1.7)	1.0
- Fluoroquinolones	25 (11.8)	12 (14.6)	9 (12.9)	4 (6.7)	1.0
- Tetracyclines	2 (1.0)	0	1 (1.4)	1 (1.7)	0.97
- Metronidazol	11 (5.2)	3 (3.6)	3 (4.3)	5 (8.3)	0.99
- Clindamycin	7 (3.3)	1 (1.2)	1 (1.4)	5 (8.3)	0.88
- Vancomycin	20 (9.4)	6 (7.3)	5 (7.1)	9 (15.0)	0.99
- Fluconazole	1 (0.5)	1 (1.2)	0	0	1.0
Characteristics of antimicrobial therapy:					
- No. of empirical combinations	45 (21.2)	14 (17.1)	11 (15.7)	20 (33.3)	0.06
- No. of adequate empirical antimicrobial therapy	190 (89.6)	74 (90.2)	63 (90.0)	53 (88.3)	0.84
- No. of escalations of antimicrobial therapy	36 (16.9)	9 (10.9)	14 (20.0)	13 (21.6)	0.17
- No. of de-escalations of antimicrobial therapy	30 (14.2)	12 (14.6)	4 (5.7)	14 (23.3)	0.02***
- No. of patients with subsequent oral switch	55 (25.9)	25 (30.5)	21 (30.0)	9 (15.0)	0.06
No. of patients receiving supportive therapy	87 (40.7)	15 (18.1)	28 (40.0)	44 (72.1)	< 0.01*^,^**^,^***
Types of supportive therapy:					
- Hemodynamical support	47 (22.0)	0	2 (2.9)	45 (73.8)	< 0.01**^,^
- Mechanical ventilation	40 (18.7)	2 (2.4)	10 (14.3)	28 (45.9)	< 0.01*^,^**^,^***
- Renal replacement therapy	6 (2.8)	0	1 (1.4)	5 (8.2)	0.01**
- Any corticosteroid therapy	33 (15.4)	10 (12.0)	14 (20.0)	9 (14.8)	0.39
- Human albumin	17 (7.9)	2 (2.4)	3 (4.3)	12 (19.7)	< 0.01**^,^***
- Red blood cell transfusion	43 (20.1)	5 (6.0)	12 (17.1)	26 (42.6)	< 0.01**^,^***
- Thrombocyte transfusion	15 (7.0)	1 (1.2)	2 (2.9)	12 (19.7)	< 0.01**^,^***
Duration of supportive therapy (days, median ± IQR, min–max):					
- Hemodynamical support	4.0 ± 7.0 (1–30)	0	2.0 ± 1.0 (1–3)	4.5 ± 8.3 (1–30)	0.11
- Mechanical ventilation	7.0 ± 8.0 (1–27)	12.5 ± 4.5 (8–17)	7.0 ± 5.5 (1–20)	7.0 ± 10.5 (1–27)	0.66
- Renal replacement therapy	7.5 ± 8.8 (1–15)	0	1 (1–1)	10.0 ± 9.0 (4–15)	0.33
- Any corticosteroid therapy	5.0 ± 6.0 (1–20)	6.0 ± 5.0 (3–14)	5.0 ± 3.0 (2–20)	6.0 ± 9.5 (1–18)	0.68
- Human albumin	4.0 ± 2.0 (1–10)	3.5 ± 1.5 (2–5)	5.0 ± 1.5 (3–6)	4.0 ± 2.0 (1–10)	0.73

^a^ All data are reported in absolute numbers and relative percentages (n, %), unless otherwise specified

^b^ Overall duration of empirical and targeted antimicrobial therapy

* *p* < 0.05 for comparison between the groups sepsis and severe sepsis

** *p* < 0.05 for comparison between the groups sepsis and septic shock

*** *p* < 0.05 for comparison between the groups severe sepsis and septic shock

### Clinical outcomes

Clinical outcomes are described in Table 3. In the cohort, in-hospital mortality was 14.0% (30/214) and peaked among septic shock patients (42.6%). In addition, these patients were admitted to the ICU in significantly higher frequencies (57.4%). The rates of LOS and ICU LOS did not differ statistically between subgroups. Bacteraemia was detected in 50.9% (109/214) of cases, and source control could be executed in 18.2% (39/214). Results of the univariate and multivariate analysis of parameters associated with in-hospital mortality are shown in Table 4. The absence of fever or the presence of shock at diagnosis, male gender and the need for ICU admittance were selected as risk factors, and source control and de-escalation of empirical antimicrobial therapy were selected as protective factors.

**Table 4:** Predictors of in-hospital all-cause mortality among adult patients with community-acquired sepsis by survival group

Parameters^a^	Survival (*n* = 184)	Exitus (*n* = 30)	Univariate		Multivariate	
			OR (95% CI)	*p* value	OR (95% CI)	*p* value
Age (years, median ± IQR, min–max)	58.4 ± 34.0 (18–93)	66.7 ± 21.0 (26–90)	0.98 (0.96–1.01)	0.06	–	–
Male gender	76 (41.3)	16 (53.3)	1.62 (0.75–3.52)	0.22	6.35 (1.24–32.51)	0.03
Arterial hypertension	109 (59.2)	23 (76.7)	2.22 (0.9–5.26)	0.09	–	–
Chronic heart disease	78 (42.4)	16 (53.3)	1.56 (0.72–3.45)	0.27		
Chronic lung disease	25 (13.6)	9 (30.0)	2.7 (1.11–6.66)	**0.03**	–	–
Chronic kidney disease	43 (23.4)	7 (23.3)	1.0 (0.4–2.49)	0.99		
Chronic liver disease	44 (23.9)	11 (36.7)	1.85 (0.81–4.17)	0.14		
Diabetes mellitus	52 (28.3)	9 (30.0)	1.09 (0.13–2.56)	0.85		
Malignancy	20 (10.9)	2 (6.7)	0.59 (0.13–2.63)	0.49		
Corticosteroid use	14 (7.6)	3 (10.0)	1.35 (0.36–5.0)	0.65		
Immunosuppression	46 (25.0)	11 (36.7)	1.72 (0.76–3.85)	0.19		
Cerebrovascular disease	44 (23.9)	14 (46.7)	2.78 (1.27–6.25)	**0.01**	–	–
Intravenous drug use	7 (3.8)	0	n.a.	n.a.		
Excess alcohol use	33 (17.9)	12 (40.0)	3.0 (1.33–7.14)	**0.01**	–	–
No. of comorbidities (per patient, median ± IQR, min–max)	3.0 ± 3.0 (0–8)	4.0 ± 2.0 (0–7)	1.32 (1.06–1.61)	0.08	–	–
Charlson score (median ± IQR, min–max)	4.0 ± 5.3 (0–12)	6.0 ± 4.8 (0–9)	1.11 (0.98–1.28)	0.09	–	–
Absence of fever at diagnosis	14 (7.6)	8 (26.7)	4.42 (1.67–11.71)	**0.01**	7.35 (1.1–51.8)	0.04
Presence of shock at diagnosis	35 (19.0)	26 (86.7)	27.78 (9.09–83.3)	**< 0.01**	33.93 (6.72–141.47)	< 0.01
Identifiable source of sepsis	163 (88.6)	27 (90.0)	1.16 (0.32–4.17)	0.82	–	–
Source control	36 (19.6)	3 (10.0)	0.46 (0.13–1.59)	0.22	0.09 (0.01–0.63)	0.02
Rate of bacteraemia	92 (50.0)	17 (56.7)	1.29 (0.59–2.86)	0.49	–	–
Need for ICU admittance	36 (19.6)	22 (73.3)	11.11 (4.55–25.0)	**< 0.01**	8.39 (1.67–42.05)	0.01
Need for supportive therapy	63 (34.2)	24 (80.0)	7.69 (2.94–20.0)	**< 0.01**	–	–
Duration of antibiotic therapy (days, median ± IQR, min–max)	11.0 ± 4.0 (1–50)	6.5 ± 12.8 (1–30)	1.09 (1.02–1.19)	0.01*	n.a.	
Adequate empirical antimicrobial therapy	164 (89.1)	26 (86.7)	0.71 (0.22–2.27)	0.56	–	–
Escalation of antimicrobial therapy	28 (15.2)	8 (26.7)	2.0 (0.81–5.0)	0.13	–	–
De-escalation of antimicrobial therapy	27 (14.7)	3 (10.0)	0.64 (0.18–2.27)	0.48	0.14 (0.02–1.06)	0.04
Empirical antimicrobial combinations	37 (20.1)	8 (26.7)	1.42 (0.59–3.45)	0.43		
Penicillins as empirical choice	22 (12.0)	4 (13.3)	1.13 (0.36–3.57)	0.83		
Cephalosporins as empirical choice	129 (70.1)	24 (80.0)	1.69 (0.74–4.35)	0.38		
Carbapenems as empirical choice	13 (7.1)	3 (10.0)	1.47 (0.39–5.56)	0.57		

^a^ All parameters are reported in absolute numbers and relative percentages (n, %), unless otherwise specified

* The parameter was not included in the final model as co-linearity was not proven by the Box-Tidwell test (*p* < 0.05)

n.a. Not applicable.

## Discussion

### Present study

In this retrospective observational cohort study, we analysed the clinical and microbiological characteristics and outcomes of community-acquired sepsis (CAS) among adult patients admitted to a single national center during a 1-year period. As of December 2018, this is the first study done on the subject in Hungary.

Firstly, we conclude that CAS is a notable entity with an estimated annual incidence of 7.2 cases per 100.000 inhabitants, which roughly translates to 3 septic patients per 100 new hospital admittances from the community. Severe sepsis and septic shock accounted for the majority (131/214, 61.2%) of cases. Older patients were usually treated for chronic cardiopulmonary diseases, diabetes mellitus or other forms of immunosuppression. Septic shock was associated with cerebrovascular and chronic liver disease, probably due to the overall poor functional status of these patients. Considering demographic data, there was a slight tendency towards female dominance in the cohort.

Secondly, we think that certain clinical and microbiological characteristics might be representative of CAS. The source was urogenital or abdominal infection in approximately half of the cases (105/214, 49.1%), and abdominal infections were more likely to progress to septic shock. The latter suggests that higher alertness from attending physicians might be beneficial for patients with intra-abdominal sepsis, and an abdominal source should be suspected more emphatically in case of a septic shock without an obvious focus. We found that higher body temperature was noted frequently (192/214, 89.7%), but the presence of fever did not correlate with severity of sepsis. With an exceptionally high rate of blood culture sampling at our institution, bacteraemia could be identified identified in every second case. These numbers might serve as major implicatons for clinical care. We should note that 43% (92/214) of cases were caused by three significant pathogens, *E. coli, S. aureus* and *S. pneumoniae*. We also emphasize that the causative organisms isolated were likely to be poli-sensitive in vitro, and in only 1.9% (4/214) were MDR isolates (ESBL producing *E. coli*, MRSA) cultured. This low rate of resistance should be considered when choosing an empirical regime for CAS, as it may facilitate the avoidance of unnecessarily broad-spectrum antibiotics (eg. carbapenems) in certain clinical scenarios. In addition, empirical antibiotic therapy was adequate in the majority of cases (190/212, 89.6%), and the initial use of cephalosporins or penicillins was not found to be associated with poorer outcome.

Finally, CAS requires significant healthcare expenditures, and upholds a complex burden of disease, especially for patients with septic shock. In our cohort, in-hospital all-cause mortality was 14.0% (30/214),and every fourth patient required admission to the ICU (58/214, 27.1%). Both outcomes were statistically more prevalent among septic shock cases (42.6 and 57.4%). Patients who are at a higher risk of death might be identified earlier by contributing independent risk factors, such as the presence of shock (OR 33.93, 6.72–141.47) or the absence of fever (OR 7.35, 1.1–51.8) at diagnosis, or male gender (OR 6.35, 1.24–32.51) and the need for ICU admittance (OR 8.39, 1.67–42.05). The median LOS and the duration of antimicrobial therapy were both longer than 1 week, irrespective of sepsis severity. The source control (OR 0.09, 0.01–0.63) and de-escalation of initial empirical antimicrobial therapy (OR 0.14, 0.02–1.06) were identified as protective factors against in-hospital mortality. According to our findings, de-escalation was done in 14.2% (30/214) of cases, most frequently in a septic shock (23.3%, 14/61). This frequency of de-escalation does not necessarily reflect an incorrect strategy of therapy, but rather the appropriately wide-spectrum of the initial antimicrobial agent(s). Also, patients surviving long enough to receive microbiological results are usually subjected to de-escalation at our institution. Source control was performed in 18.2% (39/214), as this is a necessary action during management if feasible.

### Previous studies

Differences among morbidity estimates of community-acquired sepsis were noted by prior investigations in population-based studies. A prospective cohort study including 220 patients from Norway by *Nygård* et al. found an annual incidence of 0.5 cases per 1000 inhabitants and 2.2 cases per 1000 hospital admissions [16]. In contrast, *Henriksen* et al. calculated an incidence of 7.31 per 1000 person-years with sepsis of any severity in a Danish survey analysing 8358 consecutive admissions to one emergency department, while a national population-based cohort study conducted by *Wang* et al. with data from 30,239 adults living in the USA documented an incidence of 8.3 per 1000 person-years for first episode of sepsis, and 6.2 per 1000 person-years for first episode of severe sepsis during a 6-year follow-up time [17, 18]. Several previously conducted studies also illustrate the similarities of demographics and the burden of chronic diseases, both increasing the overall vulnerability for CAS [16, 19–22]. *Wang* et al. detailed that 81% of patients were ≥ 60 years old with 52.4% of males in a cohort of 970 septic adults, and similarly, in the previous study by *Nygård* et al., there was also a slight (53.2%) male dominance with 65.5% of patients who were ≥ 60 years old [16, 19]. Chronic illnesses, most frequently cardiopulmonary and renal diseases, diabetes mellitus, cancer, immunosuppression, and alcoholism-related conditions were identified as independent risk factors for hospitalization due to CAS in a case–control study, examining data of 1713 septic patients by *Henriksen* et al. [20]. Additionally, compared to sepsis-free cohorts taken from the population-level, adults with CAS had higher rates of preadmission comorbidity burden in two separate studies [19, 22].

Considering the probable pathogen spectrum of CAS, a gross concordance could be observed among recent studies, independently of geographical locations or examined populations. As reported by several investigators, the main isolated organism is *E. coli*, accounting for 25–30% of cases, followed by *S. aureus*, *S. pneumoniae* and *K. pneumoniae*, and lastly, other species of *Streptococci* and Gram negative bacteria. In addition, *P. aeruginosa* and *C. albicans* were not found to be prevalent agents of CAS [16, 21, 23–27]. Suprisingly, in an analysis of 7618 Danish patients between 1994 and 2013 by *Søgaard* et al., trends in the isolation frequencies of common pathogens seemed to be stable during two decades, with the notable exception of *S. pneumoniae*, probably due to the widespread use of vaccination [27]. Based on literature data, a microbiological etiology could be identified in 61–77%, while bacteraemia rates accompanying CAS vary between 13 and 37% [16, 21, 24–26]. Prior results also show that multidrug-resistant isolates are not highly prevalent among community-acquired sepsis. In the study of *Nygård* et al., only 2 cases of ESBL producing Gram-negative bacilli, and no MRSA were detected among community cases. The latter was also confirmed by *De Bus* et al. [16, 23]. *Retamar* et al. examined 341 episodes of community-onset bloodstream infections, and they found that 6.3% were caused by MDR isolates, from which 3.8% were ESBL producers. Again, MRSA was not reported as a causative agent [28]. However, we note that some authors document MDR organisms as possible etiologies at higher rates, but these results should be interpreted with caution. *Lim* et al. published a relatively higher prevalence of MDR Gram-negative bacteria (9–26%) and MRSA (20–30%) causing community-onset bloodstream infections in a 1:1 matched case–control study with 360 patients during a 10-year period, but risk factors identified in association with this microbiological outcome reflected significant healthcare exposure (eg. long-term care facility resident, immunosuppression, chronic wound care, recent surgery and antibiotic exposure) [29]. Additionally, a recent retrospective cohort study conducted in the USA reported an outstanding prevalence of MRSA (41.2%) as etiology among 1677 community-associated bloodstream infections, but predictors of resistance were also in connection with healthcare exposure (eg. recent hospitalization and presence of malignancy) [30]. A multicentric observational study of 682 community-onset *Enterobacteriaceae* bloodstream infections done by *Zahar* et al. pointed out that despite 8.5% being caused by ESBL producing bacteria, almost two-thirds of these infections were actually healthcare-acquired, and the main risk factor of community-onset ESBL-positive bacteraemia was a hospital stay within one year. The authors concluded that true community-acquired bloodstream infections with ESBL-positive isolates remain rare [31]. In summary, CAS episodes caused by MDR pathogens should be acknowledged, but a dominant proportion of these cases is probably community-onset, healthcare-acquired sepsis. We emphasize that a detailed patient history and consideration of local antimicrobial resistance rates are imperative in the accurate identification of such episodes and the choice of empirical therapy.

Contrary to our findings, previous studies noted pneumonia as the most common source for CAS, with incidence rates ranging from 30.2 to 65.8%, followed by genitourinary (14.1–26.4%), abdominal (10.7–24.2%) and soft tissue infections (8.1–12.3%) [11, 16, 19, 21, 22, 26]. Additionally, the study conducted by *Søgaard* et al. suggests that frequencies of different septic sources among CAS patients may shift over long observational periods. For example, incidence of respiratory and abdominal sources decreased by approximately 6–6%, while the rate of unknown sources increased steadily by 14.9% during a 19 year period [27]. The vast healthcare burden is reflected in clinical outcomes of CAS as well. According to medical literature, the overall mortality of CAS during hospitalization is reported between 25 and 51.6% of cases, with 14.4–16.7% measured at 30 days [11, 16, 21, 24–27]. Different organ and metabolic dysfunctions and higher APACHE II, SAPS II or SOFA scores, clinically coresponding to advanced stages of sepsis with multi-organ involvement, are independently associated with in-hospital death. Some other risk factors were malignancies and cardiovascular diseases, microbiological characteristics such as non-identified etiology and blood culture positivity, and certain sources of infection (endocarditis, abdominal and urinary tract infections), while important modifiable risk factors were delay in administration and inadequacy of empirical antimicrobial agents [11, 16, 21, 26, 32]. The need for ICU admission ranged from 0.3 to 48.9%, with proportionately higher rates among patients with shock, as they demand the most advanced techniques of medical support. ICU LOS was moving between 10 and 15 days, while non-ICU LOS was 9–12 days in most studies [11, 16, 22, 24]. Our key observations are highly comparable and might be externally validated by these clinical outcomes.

### Limitations

Our study has several limitations. Firstly, the single-centered nature could bias towards more positive clinical outcomes, and the heterogenity of patients may be underestimated. Some patients may have been transported to other hospitals in the area or discharged with incorrect ICD–10 codes, lowering the pool of eligible patients and estimated annual incidence. Despite using pre-defined exclusion criteria, some unaccounted overestimation might have arisen from the inclusion of community-onset, healthcare-acquired sepsis cases. Data collection might have been affected by documentational bias. Another limitation for interpretation could originate from the subjective intuition of clinicians affecting their choices (bias by indication). Some over-fitting might have arisen during logistic regression, limiting its predictive value. Lastly, as with all retrospective studies, correlations and risk factors described here should be validated in a prospective and well-powered study. In spite of limitations, we believe that our study could help to deepen our knowledge of CAS by providing current insights among Hungarian patients. We hope that this study might pilot upcoming prospective, multi-centered studies on the subject.

## Conclusions

We aimed to evaluate the characteristics and outcomes of community-acquired sepsis in an adult cohort of a high-influx national medical center. In our study, CAS was found to be a prevalent entity with characteristic sources and documented organisms of infection. A relevant proportion of cases were caused by one of 3 main bacteria, and most isolates showed in vitro poli-susceptibility, so a sparing approach with broader spectrum antibiotics might be considered when choosing empirical therapy. In-hospital mortality was high in severe cases, but patients at greater risk for a poor outcome could be identified earlier by contributing factors. More prospective studies on the subject are warranted.

## Data Availability

The datasets used and/or analysed during the current study are available from the corresponding author on reasonable request.

## Abbreviations

ACCP: American College of Chest Physicians
APACHE II: Acute Physiology and Chronic Health Evaluation II
CAS: Community-acquired sepsis
CDC: Centers for Disease Control and Prevention
CMV: Cytomegalovirus
EBV: Epstein––Barr-virus
ESBL: Extended-spectrum beta-lactamase
HSV1: Herpes simplex virus-1
ICD–10: International Classification of Diseases, 10th edition
ICU: Intensive care unit
IQR: Interquartile range
LOS: Length-of-stay
MALDI/TOF–MS: Matrix-assisted laser desorption/ionization / time-of-flight mass spectrometry
MDR: Multidrug-resistant
MRSA: Methicillin-resistant S.aureus
PCR: Polymerase chain reaction
SAPS II: Simplified Acute Physiology Score II
SCCM: Society of Critical Care Medicine
SD: Standard deviation
SIRS: Systemic inflammatory response syndrome
SOFA: Sequential Organ Failure Assessment
